# Derived generalization of attentional bias for laboratory-induced threat: Yes but

**DOI:** 10.3389/fpsyg.2022.1004157

**Published:** 2022-12-15

**Authors:** Sonsoles Valdivia-Salas, Andrés Sebastián Lombas, Ginesa López-Crespo, Pablo J. L. Zaldivar

**Affiliations:** Department of Psychology and Sociology, University of Zaragoza, Zaragoza, Spain

**Keywords:** attentional bias, derived generalization, transfer of functions, spatial cueing task, adults

## Abstract

There is laboratory evidence that fear conditioning underlies the emergence of attentional bias (AB) for threat. Our main objective was to test, for the first time, whether derived or symbolic responding contributes to the generalization of AB across non-conditioned stimuli. Participants were all university students (*N* = 86) with no pre-existing conditions. We first employed an exogenous cueing paradigm with two color slides (i.e., A1 or to-be CS+, and A2 or to-be CS−) serving as cues, and loud white noise serving as unconditioned stimulus during conditioning trials. We then employed a match-to-sample procedure to establish a derived equivalence relation between color A1 and arbitrary shape C1 as well as between color A2 and arbitrary shape C2. Next, we investigated the transfer of AB across non-conditioned stimuli: participants performed the same spatial cueing task with non-conditioned C1 and C2 stimuli serving as cues. Results replicated previous findings on the conditioning basis of AB, and most importantly, showed preliminary evidence of AB transfer: those participants who appraised C1 and not C2 as a signal of impending noise showed AB toward C1. This is the first laboratory demonstration that AB may generalize to stimuli physically unrelated to directly conditioned threats. Unfortunately, the small number of participants showing this effect calls for cautious considerations.

## 1. Introduction

Attentional Bias (AB) is traditionally regarded as the hypervigilance to cues which signal impending danger from the environment (Beck et al., 1985). AB has been described in pain, depression, fear, and anxiety, and also in normal motivational states such as hunger (Bar-Haim et al., 2007; Yiend, 2010; Crombez et al., 2013). In this paper, we focus on AB toward threat because of its prevalence among anxiety disorders (e.g., Bar-Haim et al., 2007).

Several models of attention to threat propose that directing attention to imminent threat is a phylogenetically selected mechanism that is part of normal cognitive functioning (e.g., Mathews and Mackintosh, 1998; Mogg and Bradley, 1998; Eccleston and Crombez, 1999), i.e., AB helps the organism detect danger in the environment and produce a prompt and effective response. Notwithstanding, anxious individuals seem to be overly sensitive to and biased toward threat-related information (Bar-Haim et al., 2007). Such is the prevalence of AB toward threat among anxiety disorders, that several cognitive accounts regard it as a vulnerability factor for the emergence, maintenance, and exacerbation of dysfunctional fear and anxiety. Still, the nature of the causal relation between AB and fear/anxiety is an issue of theoretical controversy. Briefly, whereas some models postulate AB as the cause of fear and anxiety, others argue the opposite causal relation. Empirical evidence supports both views, complicating the drawing of firm conclusions (for an extended review of this controversy, see Van Bockstaele et al., 2013).

In an attempt to provide answers about how AB develops, some researchers have explored the conditions that turn an otherwise neutral event into a threatening stimulus. Classical conditioning has been useful in addressing this question. There is evidence that after repeated pairings of a neutral stimulus (to-be CS+, conditioned stimulus) with a threatening stimulus (UCS, unconditioned stimulus), the former produces AB in laboratory settings (e.g., Crombez et al., 2013; Valdivia-Salas et al., 2014). Relevant to this paper is the research employing the modified version of the spatial cueing task. In this task, participants are asked to detect a visual target that is presented at the left or right side of a fixation cross. This visual target is preceded by a neutral cue stimulus that is flashed for a short interval at the same (valid trials) or opposite (invalid trials) spatial location. Detection times are usually shorter in valid trials than they are in invalid trials, which is known as the cue validity effect. In the modified version of this paradigm the cues may have a threatening meaning (e.g., a CS+), which allows the investigation of the engagement with (valid trials) and difficulties to disengage from (invalid trials) a threatening cue. Attentional engagement is the tendency for attention to focus on initially distal negative information, probably contributing to anxiety reactivity. Attentional disengagement, on the other hand, is the tendency for attention to remain focused on negative information, and it is believed to contribute to anxiety perseveration (Grafton and MacLeod, 2014).

Research using the modified paradigm has shown both facilitated engagement with and impaired disengagement from a laboratory-induced threatening cue (e.g., Notebaert et al., 2011). More specifically, after fear-conditioning, healthy individuals are faster in detecting a target when it is validly cued by a threatening stimulus as opposed to a neutral stimulus; and slower in detecting a target when it is invalidly cued by a threatening stimulus as opposed to a neutral stimulus. Overall, these studies point to classical conditioning as a plausible avenue to develop AB toward threat. It is unlikely, however, that classical conditioning could explain the generalizability of AB shown by clinical patients. For instance, it is common to find patients who continue to show AB long after the conditioning experience took place, who show AB to stimuli different to those that were conditioned, or who, in fact, cannot remember any direct conditioning episode. We argue that besides direct learning, learning through language may further account for the complexity of AB in clinical populations.

Within a functional-contextual approach to language, “languaging” is the ability to infer or *derive* relations between stimuli that were never directly learned (Hayes et al., 2001). This ability has been consistently shown in the experimental context by using the matching to sample (MTS) paradigm. Here, the sample stimulus, say A1 (usually an arbitrary visual stimulus) is presented at the top of the screen along with three comparison stimuli, say B1, B2, and B3 (usually three visual arbitrary stimuli sharing no physical properties with the sample stimulus) at the bottom of the screen until the participant reliably picks comparison B1 in the presence of sample A1. Then, B1 is set as the sample stimulus and is followed by three different comparison stimuli, say C1, C2, and C3, until the participant reliably picks comparison C1 in the presence of sample B1. When the relations A1–B1 and B1–C1 are established, their position as sample and comparison stimuli is reversed. In such conditions, humans have no difficulty selecting A1 in the presence of B1, B1 in the presence of C1, C1 in the presence of A1, and most importantly, A1 in the presence of C1. This is a remarkable achievement and is called derived relational responding (Hayes et al., 2001).

The most interesting effect associated with derived relational responding is transfer or transformation of functions, due to its clinical implications. Transfer of functions refers to the indirect acquisition of functions by stimuli that participate in relations with other stimuli (e.g., Dougher, 1998; Dougher, 2021). In the sample above, if A1 acquired reinforcing functions through direct conditioning, then B1 and C1 would acquire similar functions without needing explicit conditioning. We then say that A1, B1, and C1 form an equivalence class or relational frame of coordination (Sidman, 1994; Hayes et al., 2001), and the reinforcing properties of A1 have transformed the functions of B1 and C1 by derived means (Dougher, 1998).

Transformation of functions has been shown with a number of stimulus functions, including discriminative, respondent, consequential, contextual, and avoidance (e.g., Dougher et al., 2007; Rodríguez Valverde et al., 2009; Hooper et al., 2010; Gil et al., 2012; Valdivia-Salas et al., 2013). There is no evidence, however, that transformation of functions may be involved in the generalizability of AB toward threat. The present study aims to fill this gap.

Our main objective was to test, for the first time, whether laboratory-induced AB transfers across equivalent stimuli. During the first part of the study, we employed similar procedures as in previous studies on the conditioning basis of AB for threat (e.g., Koster et al., 2004). Once participants showed AB toward directly conditioned color slide A1 and color slide A2 during the modified version of the spatial cueing task; we employed a MTS procedure to establish a derived equivalence relation between A1 and arbitrary shape C1, and between A2 and arbitrary shape C2. Next, we investigated the transfer of AB across non-conditioned stimuli: participants performed the modified version of the spatial cueing task, but with non-conditioned C1 and C2 stimuli now serving as cues. If the response pattern to C stimuli were similar to that to A stimuli, then this would prove that transfer of functions may be one mechanism involved in the generalization of AB toward otherwise neutral stimuli.

## 2. Materials and methods

### 2.1. Participants

University students were recruited through online announcements and personal contact. A total of 86 first year undergraduate students (87.2% female; mean age = 19.03 years, *SD* = 3.4) participated in the study and received course credit in return. All of them had normal or corrected-to-normal vision, and reported no color-blindness, psychiatric or medical condition that might pose a risk for the procedures implemented (e.g., anxiety, epilepsy). None of them were familiar with the tasks. Before the experiment proper, the experimenter described the general procedure, and participants provided a written informed consent. Upon completion of the study, participants were fully debriefed.

### 2.2. Stimuli and tasks

The experiment was conducted in a dimly lit, sound attenuated chamber (3 m × 1.5 m) equipped with a desk and a DELL desktop computer. Participants sat approximately 60 cm from the computer screen, and were in visual and auditory contact with the experimenter through a two-way mirror. All tasks were computerized: the computer ran a customized Visual Basic program during matching to sample procedures, and an e-Prime program during spatial cueing tasks.

#### 2.2.1. Match-to-sample task

**Figure 1:**
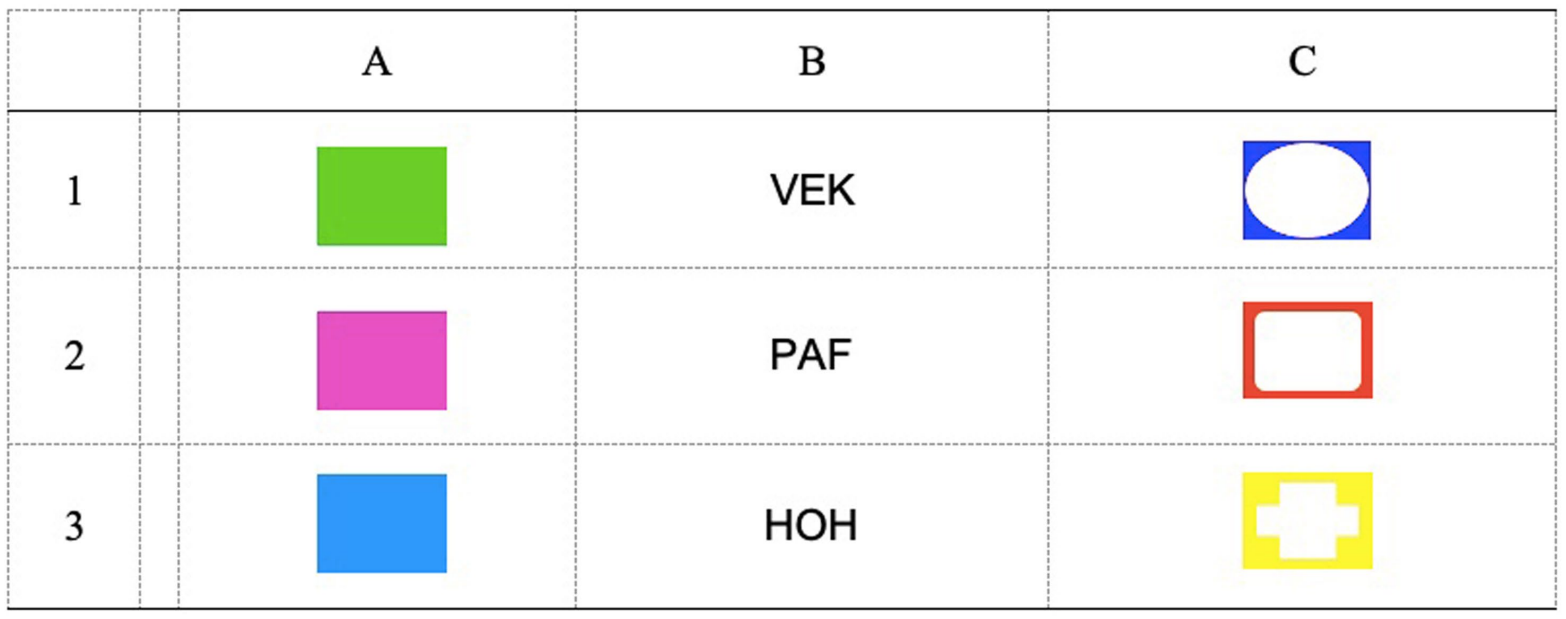
Stimuli used during the experimental tasks.

A linear Match-to-sample (MTS) procedure was used to train and test the formation of two 3-member equivalence classes (A1–B1– C1/A2–B2–C2). Alphanumerical labels are used here for descriptive purposes only. As depicted in Figure 1, the A stimuli, which served as samples during training, were colored rectangles (green, pink, and blue). The B and C stimuli, which served as comparisons during training, were nonsense syllables (VEK, PAF, HOH) and colored shapes (blue circle, orange square, and yellow cross), respectively. The assignment of colored rectangles as A1 and A2 was counterbalanced across participants, same as the assignment of colored shapes as C1 and C2. Thus, Figure 1 depicts a particular assignment of alphanumerical labels that was true in only some of the participants. During each trial, the sample was presented at the top center of the screen. After a 2000 ms delay, three comparisons appeared along the bottom until a response was emitted. No response was required to the sample stimulus. Comparisons were always from the same alpha group (e.g., B1, B2, B3). B3 and C3 served as incorrect comparisons and were used to control for responding by exclusion (see Carrigan and Sidman, 1992). The spatial location of the comparisons at the bottom varied randomly across trials. Participants selected from among the comparisons by using the keyboard. Pressing key 1 selected the comparison on the left, key 5 selected the comparison in the middle, and key 8 selected the comparison on the right. Correct and incorrect responses were followed by the written message CORRECT and WRONG, respectively, which was displayed for 1,500 ms. The next trial started after a 2000 ms intertrial interval.

#### 2.2.2. Modified version of the spatial cueing task

All stimuli were presented against a black background. On each trial, a white fixation cross appeared in the middle of the screen along with two white rectangles (4.8 cm high × 6.5 cm wide), one to its left and one to its right. The middle of the rectangles was 9.2 cm from the fixation cross. Cues and targets were presented within these two rectangles. Cues consisted of either the pink and green rectangles (A1 and A2, see Figure 1), or the blue circle and orange square (C1 and C2, see Figure 1), depending on the experimental phase, fitting in the white rectangles. The target was a black square (1 cm × 1 cm). During conditioning phases with A1 and A2 stimuli, which color functioned as CS+ and CS− was counterbalanced across participants; and the unconditioned stimulus (UCS) consisted of a 150-ms white noise burst delivered through headphones at an intensity of 100 dBA, hence aversive but not harmful (Hobbs, 1990). The noise was delivered at cue offset. A trial started with a 1,000-ms presentation of the fixation cross and white rectangles. A cue was then flashed in one of the two rectangles for 200 ms. The target appeared in one of the two rectangles 20-ms after cue offset and remained on the screen until a response was emitted, or for 2000 ms. The next trial started 1,000 ms after responding. Across phases, 75% of trials were validly cued (i.e., the target appeared at the same location than the cue) and 25% of trials were invalidly cued (i.e., the cue and the target appeared in opposite locations). The cues in each phase (either A1 and A2, or C1 and C2) were presented equally often. The order of presentation was random, with the only constraints of maximal three consecutive presentations of the same stimulus, and maximal three consecutive presentations of the target on the same location. To control for cue responding, catch trials were presented in which the cue was not followed by the target, and thus no response had to be made. As well, to control for the proper direction of the attention to the middle of the screen, digit trials were interspersed in which the fixation cross was replaced by a digit (either 6, 7, or 8; 5 mm high) for 100 ms. In these trials, participants were instructed to press the number key that matched the number on the screen.

#### 2.2.3. Evaluative ratings

At different times during the modified version of the spatial cueing procedures, participants were asked to assess the arousal, threat, and valence of conditioned and nonconditioned stimuli A1, A2, C1 and C2; as well as the UCS expectancy and valence. Both the questions and the Likert-type response options were presented in the computer screen. As an example, we present the questions for A1 stimulus. Valence: A1 seems to me: 0 (very negative) to 9 (very positive). Threat: A1 seems to me: 0 (not threatening at all) to 9 (very threatening). Anxiety: When I saw A1 on the screen, I felt: 0 (not anxious at all) to 9 (extremely anxious). UCS expectancy: I expected the US after A1: 0 (not at all) to 9 (very strongly). Participants pressed the number key of the response option that best described how they felt in relation with the stimuli and the UCS. Participants’ responses cleared the screen and led to the next question or task.

### 2.3. Procedure

**Figure 2:**
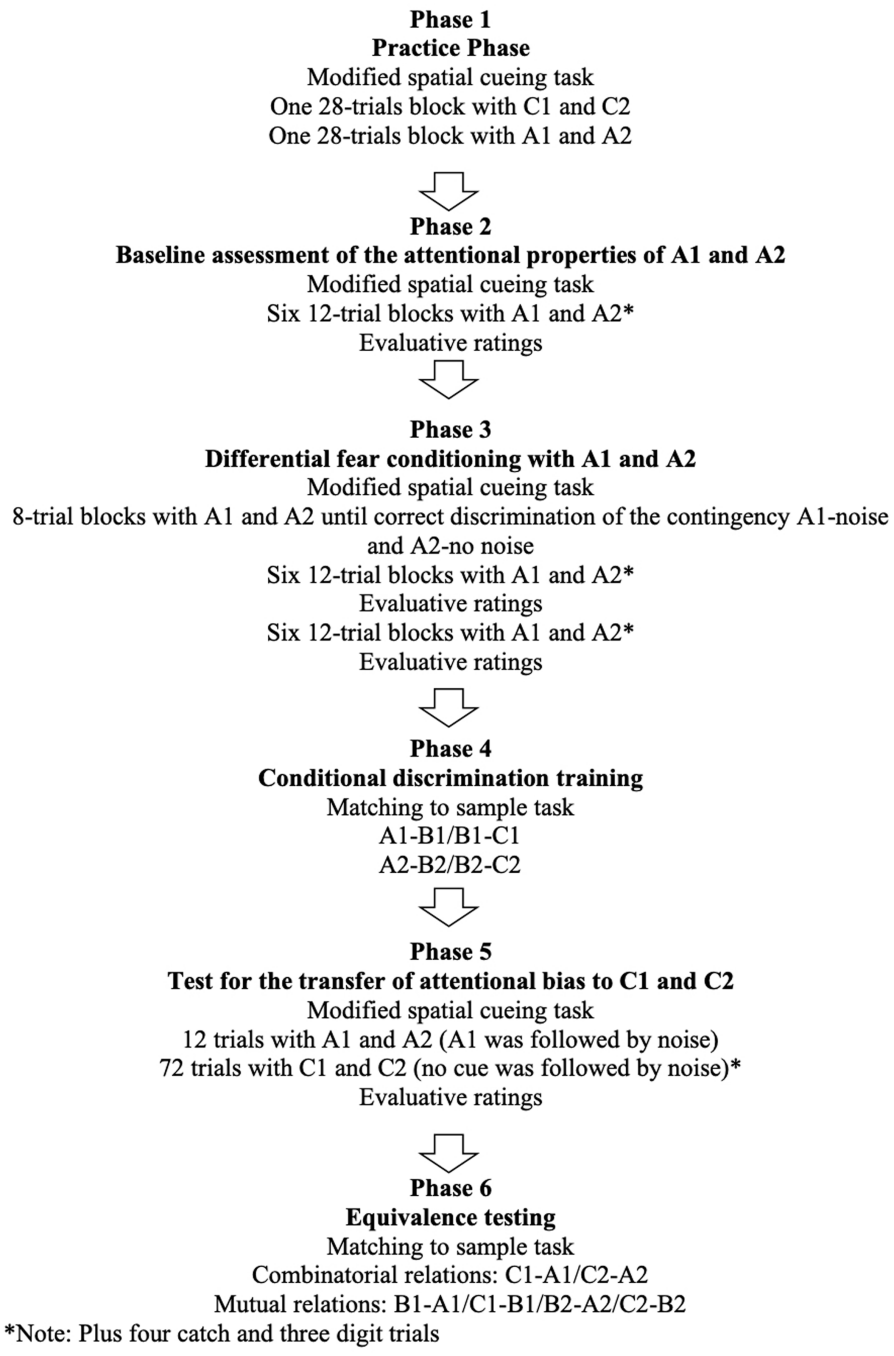
Schematic overview of the procedures employed.

After signing the informed consent, participants were escorted to the experimental room and seated in front of the computer. The experimental procedures consisted of six phases (see Figure 2), all of them conducted in a single session that lasted about 1 h. The experimenter first read the general instructions about the procedures and then left the room. The specific instructions for each task were displayed on the computer screen.

#### 2.3.1. Phase 1: Practice phase

The purpose of this phase was to get participants familiar with the spatial cueing paradigm. They were instructed to respond as quickly and accurately as possible to the location of the target by pressing the P key if it appeared on the right and the Q key if it appeared on the left. Participants practiced during two 28-trial blocks, with C1 and C2 serving as cues during the first block, and A1 and A2 serving as cues during the second block. Each block included two catch and two digit trials. This phase finished with a written message on the computer screen informing participants that the practice phase had been completed successfully and the test would start now.

#### 2.3.2. Phase 2: Baseline assessment of the attentional properties of A1 and A2

The purpose of this phase was to assess the baseline latencies to respond to the target during valid and invalid A1 (to-be CS+) trials, and valid and invalid A2 (to-be CS−) trials. Participants were reminded to respond as quickly and accurately as possible to the location of the target. This phase consisted of a total of 79 trials, including six 12-trial blocks (six A1 trials, six A2 trials), and four catch and three digits interspersed across blocks. At the end, participants rated A1 and A2 arousal, threat and valence.

#### 2.3.3. Phase 3: Differential fear conditioning with A1 and A2

This phase started with a written message on the computer screen requiring participants to put the headphones on and reminding them to respond as quickly and accurately as possible. Participants were first presented with blocks of eight spatial cueing trials in which A1 (CS+) as cue was always followed by noise (UCS), and A2 (CS−) as cue was not (four trials per stimulus). After each block, UCS expectancy was assessed. Blocks were repeated until participants rated UCS expectancy with A1 higher than 6, and UCS expectancy with A2 lower than 4. Once the contingency was correctly discriminated, two series of 79 trials were presented. Each series consisted of six 12-trial blocks (six A1 trials, six A2 trials), plus four catch and three digit trials per series. Half of the A1 trials was followed by the UCS. At the end of each series, participants rated A1 and A2 arousal, threat, and valence, as well as UCS valence and expectancy. Within blocks, trials were randomly presented. At the end of the second series, a written message on the screen informed participants that they could put the headphones off, and the instructions for the next task were presented.

#### 2.3.4. Phase 4: Conditional discrimination training

The purpose of this phase was to establish a derived relation between A1 (CS+) and C1, and between A2 (CS−) and C2. The training sequence proceeded as follows. Each new relation (starting with A1–B1) was trained until the participant emitted two consecutive correct responses. Training with the same relational pair in Class 2 (i.e., A2–B2) followed until two consecutive correct responses were produced. Subsequently, both relational pairs (i.e., A1–B1 and A2–B2) were presented in random order in blocks of four trials (two per relational pair), until completion of one block with 100% correct selections. This same sequence was repeated with the pairs B1–C1 and B2–C2. After B–C training was completed, four-trial blocks containing A–B and B–C relations (one trial per relational pair) were presented until the participant produced five consecutive blocks with 100% correct responses.

#### 2.3.5. Phase 5: Test for the transfer of AB to C1 and C2

The purpose of this phase was to examine the effect of differential fear conditioning with A1 and A2, and equivalence training, on the response latencies to respond to the target stimulus during trials cued by C1 and C2. As before, participants were instructed to respond as quickly and accurately as possible, and to put the headphones on. Given the repetitive unreinforced presentations of A1 during Conditional Discrimination Training (Phase 4) and the likely extinction of its aversive functions, the test started with one 12-trial block (six A1 and six A2 trials) using the same 50% partial reinforcement as during the Differential Fear Conditioning phase (Phase 3) for function reinstatement. This was immediately followed by a series of 79 trials including 36 C1, 36 C2, three digit, and four catch trials, distributed across nine blocks. None of the C1 or C2 presentations were followed by the UCS. At the end, participants rated C1 and C2 arousal, threat, and valence, as well as UCS expectancy and valence. A written message on the screen informed participants that they could put the headphones off, and the instructions for the next task were presented.

#### 2.3.6. Phase 6: Equivalence testing

The test included the same trial format as during Conditional Discrimination Training (Phase 4), with the exception that no feedback was provided on any trial. The four combinatorial relations (A-C and C-A) were first tested in an 8-trial block (two per relation, in random order). If participants produced a minimum of seven correct responses, the test finished. Otherwise, we proceeded to test for the four mutual relations (B-A and C-B) in an 8-trial block (two trials per relation). If participants produced at least seven correct responses, a new test of combinatorial relations was presented. Participants failing to achieve the criteria in either the mutual or the second combinatorial block were deemed as not passing the equivalence test.

### 2.4. Data analyses

PASW Statistics (SPSS) version 23.0.0 for Windows (SPSS Inc., 2011) was used for coding data and performing statistical computations. Mean reaction times (RT) were calculated per phase, stimulus, and trial type. Individual outliers, defined as response latencies that deviated three *SD*s from the individual mean latency, and errors were excluded before data analyses (5.2% of total trials). In order to determine the effect of aversive conditioning on the development of AB toward A1 but not A2, separate 2 (validity: valid and invalid) x 2 (stimulus: A1 and A2) ANOVAS were conducted for the Baseline Phase data and for the Differential Fear Conditioning Phase data. In order to examine the transfer of AB to C stimuli, an individual cueing index (ICI) was first calculated for each stimulus (i.e., A1 and A2) during Differential Fear Conditioning Phase, by subtracting the mean response latency during valid trials from the mean response latency during invalid trials. A large ICI for A1 then indicates a large difference in response latency between valid and invalid A1 trials (this means fast responses to valid trials and slow responses to invalid trials). In line with previous research on fear conditioning and AB, we expected a larger ICI for A1 (CS+) than for A2 (CS−).

Given that the transfer effects to nonconditioned stimuli can only be tested if a particular function is actually established, we used the ICI as an inclusion criterion for data analysis during the Transfer Test (Phase 5). That is, only the performance of those participants who showed larger ICI with A1 than with A2 was analyzed to test for the transfer effects to C1 and C2. The transfer of AB was analyzed by conducting a 2 (validity: valid and invalid) × 2 (stimulus: C1, C2) ANOVA.

The effect of differential fear conditioning and equivalence training on the evaluative properties of As and Cs stimuli was determined by means of separate 2 (stimulus) × 4 (evaluative domain) ANOVAS conducted for Differential Fear Conditioning and Transfer Test phases. For Baseline, a 2 (stimulus) × 3 (evaluative domain) ANOVA was conducted because UCS expectancy was not assessed.

## 3. Results

**Figure 3:**
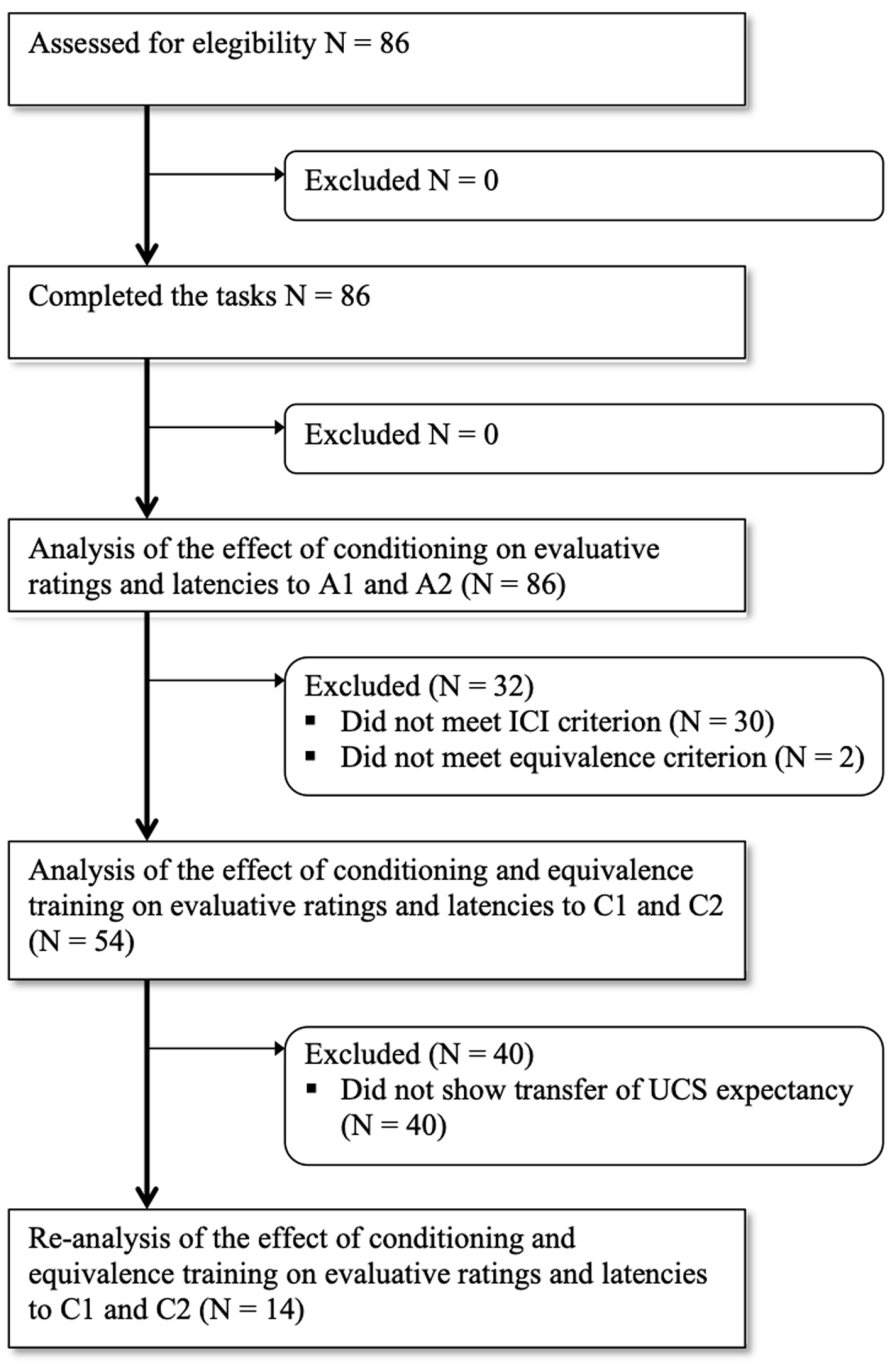
Flow of participants whose performance was analyzed through tests.

Figure 3 depicts the flow of participants whose performance was analyzed through tests.

### 3.1. Effects of differential fear conditioning with A1 and A2 on evaluative responses and response latencies to a stimuli

Table 1 shows the evaluative ratings of A1 and A2 as a function of phase. After the Baseline Phase, the ANOVA yielded a significant effect of domain, *F*(2, 170) = 10.45, *p* < 0.001, *η_p_*^2^ = 0.11; and a significant stimulus x domain interaction, *F*(2, 170) = 3.5, *p* = 0.04, *η_p_*^2^ = 0.04. Overall, threat ratings (*M* = 3.83, *SE* = 0.20) were significantly lower than arousal (*M* = 4.58, *SE* = 0.15, *p* < 0.001) and valence (*M* = 4.56, *SE* = 0.16, *p* = 0.003) ratings. The same pattern was found when analyzing the data for A1 and A2 separately. Furthermore, participants unexpectedly rated A1 as slightly but significantly more arousing (*M* = 4.80, *SD* = 1.8) than A2 (*M* = 4.36, *SD* = 1.6, *p* = 0.04), while there were no significant differences between the stimuli on the remaining evaluative domains.

**Table 1:** Average evaluative ratings and UCS expectancy to A1, A2, C1, and C2, after Baseline, Differential Fear Conditioning, and Transfer Test phases.

Test phase					
Variable	Baseline		Dif. Fear Condit.*		Transfer test**	
*M*	SD	*M*	SD	*M*	SD
Arousal						
A1 (CS+)	4.80^a^	1.77	6.89^a^	1.30		
A2 (CS−)	4.36^b^	1.60	2.56^b*^	1.33		
C1					4.79^a^	1.97
C2					3.36^b^	2.10
Threat						
A1	3.79	2.21	6.62^a^	1.71		
A2	3.86	2.11	2.23^b*^	1.38		
C1					4.93^a^	1.49
C2					3.14^b^	2.11
Valence						
A1	4.52	1.68	6.79^a^	1.35		
A2	4.59	1.78	2.95^b*^	1.42		
C1					4.86	1.17
C2					4.07	1.94
UCS expectancy						
A1			7.17^a^	1.45		
A2			1.63^b*^	0.99		
C1					5.64^a^	1.60
C2					2.14^b*^	1.56

*n = 86. **n = 14. Means with different superscripts differ at *p* < 0.05 (^a-b^) or *p* < 0.01 (^a-b*^).

After the Differential Fear Conditioning Phase, the ANOVA yielded the expected main effect of stimulus, *F*(1, 85) = 488.5, *p* < 0.001, *η_p_*^2^ = 0.85; and domain, *F*(3, 255) = 16.48, *p* < 0.001, *η_p_*^2^ = 0.16; and a significant stimulus x domain interaction, *F*(3, 255) = 22.52, *p* < 0.001, *η_p_*^2^ = 0.21, with A1 rating as significantly more arousing (mean difference = 4.33, *SE =* 0.23, *p* < 0.001), threatening (mean difference = 4.39, *SE =* 0.26, *p* < 0.001) and negative (mean difference = 3.84, *SE =* 0.27, *p* < 0.001) than A2. As well, as intended with the experimental procedures, participants showed higher UCS expectancies with A1 than with A2 (mean difference = 5.54, *SE =* 0.21, *p* < 0.001).

**Figure 4:**
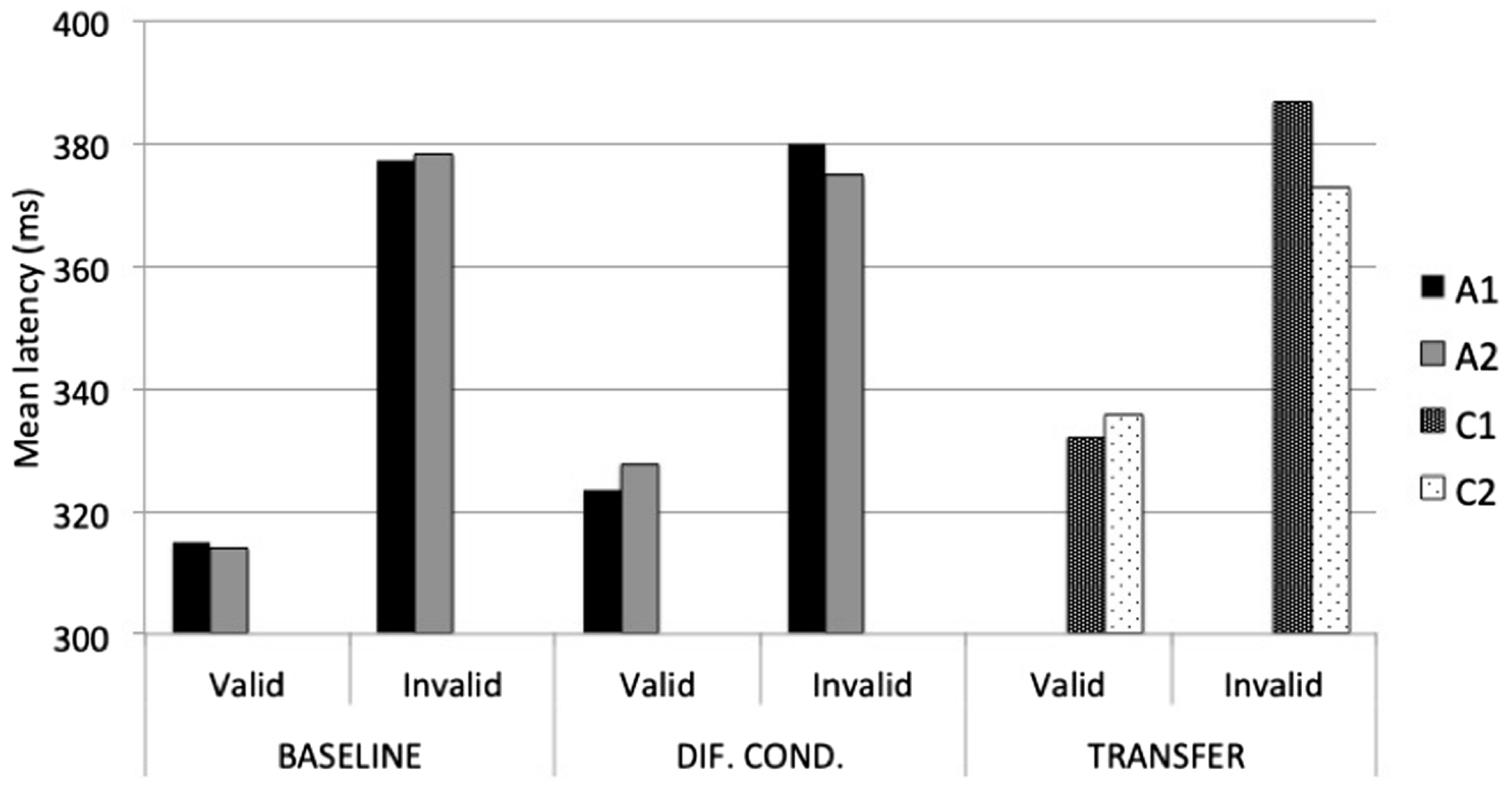
Average response latencies to A1 (CS+), A2 (CS−), C1, and C2, during Baseline, Differential Fear Conditioning, and Transfer Test phases.

The ANOVA conducted on response latencies to A stimuli during the Baseline Phase revealed the necessary main effect of validity, *F*(1, 85) = 207.47, *p* < 0.001, *η_p_*^2^ = 0.71. No other effects were significant. During the Differential Fear Conditioning Phase, the ANOVA revealed a main effect of validity, *F*(1, 85) = 220.65, *p* < 0.001, *η_p_*^2^ = 0.72; and most importantly, a validity x stimulus interaction effect, *F*(1, 85) = 8.1, *p* = 0.006, *η_p_*^2^ = 0.09. As illustrated in Figure 4, participants were slightly, although not significantly, faster responding to the target when it was cued by A1 (*M* = 323.61; *SD* = 47.34) than by A2 (*M* = 327.68; *SD* = 44.10) on valid trials (*p* = 0.07), and similarly, they were slower responding to the target when it was cued by A1 (*M* = 380.06; *SD* = 61.65) than by A2 (*M* = 374.70; *SD* = 66.76) on invalid trials (*p* = 0.07). In other words, the average difference between invalid and valid A1 trials (*M* = 56.45; *SD* = 35.13) was significantly larger than the average difference between invalid and valid A2 trials (*M* = 47.02; *SD* = 36.42), *t*(85) = 2.84, *p* = 0.006.

In order to determine who displayed an attentional bias toward the CS+, we calculated the ICI for each participant and stimulus type (i.e., A1 and A2). Out of 86 participants, 56 showed larger ICI with A1 than with A2, i.e., the difference in latency between valid and invalid trials was larger for A1 stimulus than for A2 stimulus. Only the data from these 56 participants were considered for the analysis of the transfer to C stimuli (see Figure 3).

### 3.2. Effects of differential fear conditioning with A1/A2 and equivalence training on evaluative responses and response latencies to C stimuli

All participants completed the Conditional Discrimination Training (Phase 4) successfully, taking an average of 54.3 trials (range: 34–151) to reach the mastery criterion. A necessary condition for the testing of transfer of functions from A to C stimuli is that these are in an equivalence relation with each other. This was tested during Phase 6. A total of 54 out of 56 participants reached the equivalence test criterion, that is, they correctly established A1 as equivalent to C1, and A2 as equivalent to C2. Only the data from these 54 participants entered the ANOVAs on evaluative ratings and reaction times to C stimuli (see Figure 3).

The 4 (evaluative domains) × 2 (stimuli) ANOVA on evaluative ratings of C stimuli yielded a significant main effect of domain, *F*(3, 159) = 28.12, *p* < 0.001, *η_p_*^2^ = 0.35; as well as an interaction effect, *F*(3, 159) = 4.42, *p* = 0.014, *η_p_*^2^ = 0.08. Post-hoc planned comparisons revealed that the difference in UCS expectancy between C1 and C2 was large and significant (mean difference = 0.78, *SE* = 0.27, *p* = 0.005), but the differences on the other evaluative domains were barely accountable and not significant (Arousal: mean difference = 0.04, *SE* = 0.23; Threat: mean difference = 0.09, *SE* = 0.28; Valence: mean difference = 0.15, *SE* = 0.19; *p*_s_ > 0.45). Hence, the intended main effect of stimulus was not observed.

Regarding response latencies, out of the 54 participants who met conditioning and equivalence criteria, a total of 35 (64.8%) showed larger ICI with C1 than with C2, i.e., the difference in latency between valid and invalid trials was larger for C1 stimulus than for C2 stimulus. Still, the 2 (stimulus) × 2 (validity) ANOVA on reaction times to C stimuli only revealed a main effect of validity, *F*(1, 53) = 124.1, *p* < 0.001, *η_p_*^2^ = 0.70. No other effects were significant.

To further our analyses, and given the significant difference between C stimuli in UCS expectancy, we re-analyzed the data of only those participants who also revealed higher UCS expectancy with C1 than with C2. This was the case for only 14 of the 54 participants (see Figure 3). The 4 (evaluative domains) × 2 (stimulus) ANOVA yielded a significant main effect of stimulus, *F*(1, 13) = 17.43, *p* = 0.001, *η_p_*^2^ = 0.57; and a significant interaction effect, *F*(3, 39) = 6.88, *p* = 0.008, *η_p_*^2^ = 0.35. Table 1 shows mean ratings and standard deviations on each evaluative domain. As shown, participants rated C1 as significantly more arousing (mean difference = 1.43, *SE* = 0.54, *p* = 0.02), more threatening (mean difference = 1.79, *SE* = 0.72, *p* = 0.03), and more predictive of the noise (mean difference = 3.5, *SE* = 0.52, *p* < 0.001) than C2. The only domain that did not distinguish significantly between C1 and C2 was valence. Regarding response latencies, the 2 (stimulus) × 2 (validity) ANOVA revealed a main effect of validity, *F*(1, 13) = 25.76, *p* < 0.001, *η_p_*^2^ = 0.66, and most importantly, a stimulus x validity interaction effect, *F*(1, 13) = 7.04, *p* = 0.02, *η_p_*^2^ = 0.35. As shown in Figure 4, participants were slightly, although not significantly, faster responding to a target when it was cued by non-conditioned C1 (*M* = 332.17; *SD* = 38.84) than by non-conditioned C2 (*M* = 335.74; *SD* = 49.20) on valid trials (*p* = 0.38), but they were significantly slower responding to the same target when it was cued by non-conditioned C1 (*M* = 386.64; *SD* = 58.30) than by non-conditioned C2 (*M* = 372.70; *SD* = 58.05) on invalid trials (*p* = 0.03). In other words, the average difference between invalid and valid C1 trials (*M* = 54.47; *SD* = 37.25) was significantly larger than the average difference between invalid and valid C2 trials (*M* = 36.96; *SD* = 34.47), *t*(13) = 2.65, *p* = 0.02.

We note that selecting participants on the basis of rating C1 as more arousing (*F* = 4.19), threatening (*F* = 0.85) or negative (*F* = 0.95) than C2 yielded nonsignificant effects in the ANOVAs conducted on response latencies.

## 4. Discussion

The purpose of the present study was to replicate previous findings on the conditioning basis of AB for threat as measured with the modified version of the spatial cueing task, and to examine the transfer of AB to non-conditioned stimuli. We hypothesized that after establishing (1) an attentional bias toward A stimuli, and (2) an equivalence relation between A and C stimuli, participants would also display AB toward the C stimuli. This constitutes the first laboratory attempt to demonstrate the derived transfer of AB in the laboratory. Indeed, we obtained the intended transfer effect, but the fact that it only occurred in a small portion of participants deserves cautious consideration.

The results during the Differential Fear Conditioning phase replicated previous findings, i.e., after pairing A1, but not A2, with loud white noise, participants were overall faster in responding to a target cued by A1 rather than A2 during valid trials, and slower in responding to the same target cued by A1 rather than A2 during invalid trials. A closer look at the data revealed that only 56 out of the total 86 participants (65.1%) showed this pattern of responses, according to the ICI criterion. The comparison with previous similar studies is difficult as they often report overall group effects rather than the percentage of participants meeting criteria. Still, a closer look at the data included in the meta-analysis by Crombez et al. (2013) on AB to pain-related information yields percentages ranging between 59.6 and 88.9%, our figures falling into the lower end of this range. This may be related to the fact that the UCS employed in those studies was an electrocutaneous stimulus, which has an elevated threat value (Crombez et al., 1998).

The transfer of AB for threat was tested under specific conditions that further reduced the number of participants during data analysis. This reduction of participants was the result of applying *a priori* criteria, based on theoretical issues, and also a *post hoc* criterion, based on the results obtained. First, transfer of functions refers to the derived generalization of existing functions across novel stimuli. Accordingly, transfer of functions can only be tested when the intended function is established. Thus, in our study we could only test for transfer with the 56 participants who showed larger ICI for A1 (i.e., CS+) than for A2 (i.e., CS−). Second, transfer of functions can only be tested when there is evidence that the target stimuli are part of the same equivalence class. Most of the participants (54 out of 56 participants) displayed this effect. Statistical analyses on the performance of these 54 participants revealed the absence of the expected transfer of attentional functions. In an attempt to further our analyses, we applied a third criterion, i.e., reporting larger UCS expectancies with C1 than with C2, and this reduced the sample down to 14 participants. Statistical analysis then revealed a transfer of attentional functions with a large effect size. Considering that AB for threat has been generally shown with large samples, our findings suggest that the transfer of attentional functions may be a robust phenomenon as long as certain conditions are met, namely, (1) developing AB toward threatening A1; (2) establishing C1 as equivalent to A1; and (3) showing the transfer of the UCS expectancy from A1 to nonconditioned C1. Our results prove that in humans with verbal or relational capabilities that met all those three conditions, fear conditioning with one particular stimulus may not only affect the attentional function of that stimulus but also the attentional function of equivalent non-conditioned stimuli.

Our findings illustrate how in humans, rules (Hayes et al., 1989, 2001) or propositions (De Houwer, 2009) may sometimes prevail over actual contingencies (for alternative explanations, see, e.g., Urcuioli et al., 1989; Wasserman et al., 1992). Particularly in our study: (1) during the Practice phase, participants completed the spatial cueing task with C1 and C2 serving as non-reinforced cues; and (2) during the Transfer Test phase, none of the C1 presentations were followed by the UCS. Still, some participants responded to C1 as if it were the aversively conditioned A1 stimulus. Moreover, during the transfer test we observed two interesting effects. Firstly, the average response latency with C1 during invalid trials was the slowest across test phases, and thus slower than the response latency with A1 during invalid trials after fear conditioning. Secondly, the disengagement impairment produced by C1 was significantly larger than that produced by C2, whereas there was no significant difference between stimuli in their engagement properties. These data suggest that transfer occurred for the disengagement but not for the engagement component of attention.

Some authors have hypothesized that engagement and disengagement components are independent facets of anxiety-linked attentional selectivity, and hence, they contribute to different aspects of anxiety vulnerability. Whereas attentional engagement would contribute to anxiety reactivity, attentional disengagement would contribute to anxiety perseverance (Rudaizky et al., 2012; Grafton and MacLeod, 2014). In fact, there is evidence that anxious individuals show attentional disengagement more reliably than attentional engagement (see Koster et al., 2006, for a discussion). Our data suggest that the disengagement component is more resistant to non-reinforcement than the engagement component, and thus easier to generalize across stimuli events. Research on the transfer of fear responses has shown that people may feel more physiologically activated with a non-conditioned stimulus than with a directly conditioned one, just because the former was framed as “more than” the latter (Dougher et al., 2007). The most intriguing question here is that despite the fact that C1 was established as “same as” A1, those participants who appraised C1 as a potential threat had more difficulties disengaging from C1 than from A1. This, along with the finding that attentional disengagement but not attentional engagement transferred to non-conditioned stimuli, may have important implications for the understanding of the perseverance of anxiety disorders. In spite of further replications of the present findings, our results would suggest that relational abilities may extend the range of stimuli that will be appraised as threat-relevant and produce the impaired attentional disengagement presumed to maintain and exacerbate anxiety states.

Nonetheless, the observation that transfer of attentional functions only occurred in a small subset of participants requires further consideration and discussion. First, our findings contradict the better-safe-than-sorry assumption that is often presumed to underlie easy generalization of threat (e.g., Rachman et al., 2008). It may well be that the inclusion of non-reinforced spatial cueing trials with the C stimuli during the Practice phase prevented the transfer of the UCS expectancy from A1 to C1 in some participants (Lubow, 1973). It may also be that the differences between the match-to-sample procedure and the modified spatial cueing task were large enough to keep the UCS expectancies contextual to a particular stimuli arrangement.

Second, the fact that transfer of AB was only observed in those participants who also showed transfer of UCS expectancies to non-conditioned C1 relates to the fact that transfer of functions is contextual. Contextual transfer of functions means that being in an equivalence relation does not make two or more stimuli completely interchangeable. In the typical example, we say that the written word “cake” is in an equivalence relation with the actual cake, so that if one reads the word “cake” it is very likely that they will picture the object cake. But there are specific functions of the written word and the object that do not transfer to each other. For instance, we do not *eat* the word “cake” as we do with the object, nor do we *read* an actual cake as we do with the written word. Which function transfers across stimuli depends on which contextual cues are present in a particular moment, or in non-technical terms, transfer of functions depends on context. Our findings have revealed that, in the case of healthy adults exposed to a laboratory-induced threat, the context necessary for the transfer of attentional functions may be the appraisal of a non-conditioned stimulus as a potential threat.

Our study has some limitations. First, our participants were healthy university students who were exposed to a mild aversive stimulus during a procedure held in a safe environment. Caution is needed before generalizing our results to clinical samples who might have been exposed to more life-threatening experiences. Future studies should also consider examining individual characteristics (such as trait anxiety, neuroticism, or intelligence) and tendencies (such as emotion regulation or coping) that might account for the fact that only a subsample of participants showed the transfer of evaluative and attentional functions, more specifically, the transfer of attentional disengagement. This kind of research may well serve to further our understanding of the conditions that may exacerbate or alleviate the transfer of AB, with prevention purposes.

All in all, the present findings suggest that the over generalization of AB may, given certain conditions, be an inevitable product of having relational abilities. If this effect is replicated and extended in future studies, a more thorough definition of the limiting, or pathological, properties of AB may be necessary. This new definition should place an emphasis on the processes that turn an inevitable, in certain contexts, phenomenon, i.e., AB toward non-conditioned stimuli, into a vulnerability factor for the development and exacerbation of anxiety disorders and their associated limitations.

## Data availability statement

The raw data supporting the conclusions of this article will be made available by the authors, without undue reservation.

## Ethics statement

The studies involving human participants were reviewed and approved by Comité de Ética de la Investigación de la Comunidad Autónoma de Aragón. The patients/participants provided their written informed consent to participate in this study.

## Author contributions

The procedures implemented were primarily designed and drafted by SV-S and AL. PZ administered the tasks. AL conducted data analyses. GL-C helped to draft the manuscript. All authors contributed to the revision of the draft to get the final manuscript.

## Funding

This study was supported with funds from Government of Aragon Research Council (S62_20R).

## Conflict of interest

The authors declare that the research was conducted in the absence of any commercial or financial relationships that could be construed as a potential conflict of interest.

## Publisher’s note

All claims expressed in this article are solely those of the authors and do not necessarily represent those of their affiliated organizations, or those of the publisher, the editors and the reviewers. Any product that may be evaluated in this article, or claim that may be made by its manufacturer, is not guaranteed or endorsed by the publisher.
